# Determinants in the Uptake of the Human Papillomavirus Vaccine: A Systematic Review Based on European Studies

**DOI:** 10.3389/fonc.2015.00141

**Published:** 2015-06-24

**Authors:** Victoria Fernández de Casadevante, Julita Gil Cuesta, Lourdes Cantarero-Arévalo

**Affiliations:** ^1^Section for Social and Clinical Pharmacy, Department of Pharmacy, Faculty of Health and Medical Sciences, University of Copenhagen, Copenhagen, Denmark; ^2^Infectious Disease Epidemiology, Statens Serum Institut, Copenhagen, Denmark; ^3^European Program for Intervention Epidemiology Training (EPIET), European Centre for Disease Prevention and Control (ECDC), Stockholm, Sweden

**Keywords:** human papillomavirus, vaccine, uptake, determinants, Europe, inequalities

## Abstract

**Background:**

Cervical cancer is the fourth most common cancer affecting women worldwide. Since 2006, two human papillomavirus vaccines (HPVV) have been licensed to protect women against the virus that causes cervical cancer. However, worldwide coverage remains unequal. Studies from the USA found strong evidence for differences in HPVV uptake by ethnicity and healthcare coverage. As the profile of ethnic groups and the healthcare system in the USA differ from countries in Europe where HPVV is free in most of the countries, we conducted a systematic review in order to analyze the determinants of HPVV uptake in Europe.

**Methods:**

We performed a systematic Pubmed, Scopus, and Science Direct search to find articles published from HPVV availability in European countries until April 2014. No age restriction was applied. We included all studies assessing factors associated with HPVV uptake. Uptake refers to either initiation and/or completion of the three dose vaccination program.

**Results:**

Out of the 23 eligible studies, 14 were retrospective reviews of data, six were cross-sectional surveys, and three were prospective cohort studies. Higher HPVV uptake was associated with ethnic majority populations, higher socio-economic status, regular cervical screening participation by the mother, and having received previous childhood vaccinations.

**Conclusion:**

Since the vaccine is offered for free in most of the European countries, the findings suggest that ethno-cultural and educational factors play an important role when it comes to HPVV uptake. Girls who were undervaccinated had also a lower uptake of standard childhood vaccines and mothers who were less likely to attend cervical cancer screening. This may indicate that only few parents have specific concerns with HPVV, and that preventive health care should seek ways to target these vulnerable groups.

## Introduction

The latest statistics published by the International Agency for Research on Cancer (IARC), the specialized cancer agency of the World Health Organization, shows that cervical cancer occupies the fourth position in the list of the most common cancers affecting women all over the world, preceded by breast, colorectal, and lung cancers (1). More specifically, the estimated incidence of cervical cancer was of 527,624 new cases in 2012. In the same year, cervical cancer was responsible for 265,653 deaths in the world, which constituted the fourth most common cause of cancer death worldwide (1).

Cervical cancer is often defined as a disease of disparity, because it differently affects poor and wealthy countries: at least 80% of cervical cancer deaths occur in developing countries (1). However, disparities also occur within a single country, as is the case of the USA, where Hispanic and African American women have, respectively, 2 and 1.5 times more risk of developing cancer than non-Hispanic White women (2). In Europe, the incidence and mortality rates of cervical cancer vary considerably within the region (3).

Since 2006, two human papillomavirus vaccines (HPVV) have been licensed globally, aimed at preventing cervical cancer: Cervarix^®^, a bivalent vaccine that targets papillomavirus 16 and 18, and Gardasil^®^, which additionally targets papillomavirus 6 and 11. Types 16 and 18 are responsible for around 70% of all cervical cancer cases, whereas types 6 and 11 are responsible for about 90% of anogenital warts (4). Immunization as a three-dose series against the human papillomavirus (especially before sexual onset) is recommended as primary prevention method of certain HPV infections, in order to reduce the incidence of cervical cancer and other anogenital cancer (5). However, worldwide coverage remains unequal and uptake varies widely (6).

Population-based studies (7) reporting information about HPVV uptake are helpful to identify determinants associated with poor vaccination. Hence, vaccination programs or campaigns geared toward reaching populations with low HPVV uptake can be designed to improve coverage.

A systematic review and meta-analysis (8) published in February 2013 found strong evidence for differences in HPVV initiation by factors such as ethnicity and healthcare coverage. The results were based on 27 studies, of which the majority were performed in the USA (*n* = 22), with additional studies from Canada (*n* = 2), and only three were conducted in Europe. As the healthcare system and the profile of ethnic groups in the USA significantly differ from countries in Europe, we consider it relevant to focus on studies reporting data from Europe. To our knowledge, no systematic review reporting factors associated with HPV vaccine uptake has been published to date in this specific region.

The aim of this study is to conduct a systematic review of the peer-reviewed literature in order to analyze the determinants of HPVV uptake in Europe.

## Method

### Data sources

The PRISMA guidelines (9) have been followed throughout the elaboration of this systematic review. A systematic Pubmed, Scopus, and Science Direct search was performed by the authors. The search terms were “HPV” or “human papillomavirus” AND “vaccine” or “immunization” AND “uptake” or “coverage” AND “inequalities,” “determinants,” “socio-economics,” “minority groups,” “ethnicity,” or “social background.” Those terms were also combined with “Europe,” “Eastern Europe,” “Russia.” Finally, the reference lists of the selected articles were reviewed in order to get additional references not identified via the database search.

### Eligibility criteria

We selected articles reporting HPVV uptake in females with no age restriction, and reporting at least one factor associated with vaccine uptake (either initiation, completion, or both). Articles could include register data or data from questionnaires/surveys, and no publication date filter was selected. All studies aimed at identifying and assessing factors associated with HPVV uptake were included. HPVV uptake by both routine and catch-up groups was considered. No European country was excluded. Only articles reported in English were selected. Interviews, reviews, and gray literature were excluded. Articles in which the main focus was knowledge, attitudes, or intentions to receive the HPV vaccine were also not eligible. Publications reporting the same cohorts were only included if the variables studied were different.

### Definitions

Human Papillomavirus vaccines program initiation refers to the uptake of either the first or the second dose of the three dose program recommended to get the full benefit of the vaccine.

Human Papillomavirus vaccines program completion refers to the reception of the three vaccine doses recommended to get full protection.

The routine group refers to the primary target group to receive the HPVV, which usually aims at girls that did not start their sexual life (5). Catch-up programs refer to those programs that have been created to target slightly older girls, usually up to the age of 26. The recommended age for either routine or catch-up group is determined by each country.

### Data extraction

All the relevant data were organized and extracted into the PRISMA predefined form (9). Descriptive data such as study population, study location, study time period, study design, vaccine delivery mechanism, and sample size were extracted for each study. The overall risk of bias was assessed for each study according to the sample size, and classified to be from low to high risk (<1,000 participants). (Table 1).

**Table 1:** **Descriptive characteristics of studies eligible for the review**.

Year	Authors	Country	Study time period	Study design	Study location (geographical)	Vaccine delivery mechanism	Study population	Variables adjusted	Overall risk of bias	Data extracted
2009	Rondy et al. (10)	Netherlands		Retrospective chart review	Netherlands	Healthcare setting	384,869 girls aged 13–16 years	MMR vaccination status, year of birth, country of birth, socioeconomic status	Low	OR
2012	Leval et al. (11)	Sweden	1 January, 2006 to 31 December, 2010	Retrospective cohort study	Sweden	Healthcare setting	2,209,263 women aged 10–44 years	Age	Low	RRR
2011	Widgren et al. (12)	Denmark	1 January to 31 December 2009	Register-based retrospective cohort study	Denmark	Healthcare setting	33,838 girls born in 1996	Place of origin, age of mother, number of siblings, place of residence, urban/rural (population density), MMR and DT vaccination	Low	HR
2011	Lefevere et al. (13)	Belgium	January 2007 to June 2009	Retrospective chart review	Female members of the National Alliance of Christian Mutualities Flanders, Belgium	Healthcare setting	117,151 girls aged 12–18 years	Year of birth, preferential treatment, median income neighborhood, reimbursement regime	Low	HR
2011	Giambi et al. (14)	Italy	2007–2009	Prospective cohort study	10 Local Health Units in six of Italy’s 21 Regions	Healthcare setting	1,032 women aged 18–26 years	Age, geographic area, nationality, education, employment status, marital status	Low	OR
2013	Lions et al. (15)	France	2007–2009	Retrospective chart review	South-Eastern France	Healthcare setting	105,327 girls aged 14–16 years	Age, CMU beneficiary, rural area, consultation with family physician, year of initiation, specialty of initiation, consultation with a specialist	Low	RR
2010	Rouzier et al. (16)	France	July 2007 to April/May 2009	Retrospective chart review	Paris	Healthcare setting	77,744 women aged 14–23 years affiliated to social security	–	Moderate	Pearson correlation coefficient and *p*-value
2013	Ganry et al. (17)	France	2009–2010	Retrospective chart review	Picardy, France	Healthcare setting	138,042 women aged from 14 to 23 years affiliated to social security	–	Moderate	Pearson correlation coefficient and *p*-value
2013	Lutringer-Magnin et al. (18)	France	June–August 2009	Cross-sectional survey	Rhone-Alpes region	Healthcare setting	502 women aged 14–23 years	Age, family status, hepatitis B vaccination, mother had had regular Pap Smear	Moderate	OR
2011	Blödt et al. (19)	Germany	2010	Cross-sectional survey	Six vocational schools, Berlin	Healthcare setting	259 girls and 245 boys aged 18–25 years	Years of school education, migration background, past sexual intercourse	High	OR
2013	Fisher et al. (20)	UK	2008/09 to 2010/2011	Retrospective cohort study	Three Primary Care Trusts (PCTs)/local authorities in the South West of England	PCTs	14,282 girls born between 1 September 1995 and 31 August 1998	Ethnicity, deprivation quintile, PCT/local authority responsible for delivery, program year, MMR vaccination receipt, educational setting, educational attainment	Low	OR
2008	Brabin et al. (21)	UK	February 2007	Prospective cohort study	36 secondary schools in two PCTs in Greater Manchester, UK	PCTs	2,817 schoolgirls in year 8 (12 and 13 years old)	–	Moderate	Logistic regression
2010	Kumar et al. (22)	UK	2008–2009	Retrospective chart review	152 Primary Care Trust (PCT) in England	PCTs	4,177 women	Ethnicity, childhood vaccination, cervical screening, primary care quality	Low	Regression coefficient
2011	Roberts et al. (23)	UK	2007–2008	Prospective cohort study	Two primary care trusts in Manchester	PCTs	2,817 girls aged 12–13 years	Area level deprivation, ethnicity	Low	OR
2013	Spencer et al. (24)	UK	2009–2010	Retrospective chart review	PCT in North West of England	PCTs	112,451 girls	PCT of residence, deprivation, ethnicity	Low	OR
2014	Hughes et al. (25)	UK	2008–2011	Retrospective chart review	151 PCT, England	PCT	2,493,698 girls aged 12–17 years	–	Moderate	Spearman Rank correlation coefficients and *p*-value
2013	Sinka et al. (26)	Scotland	September 2008 to August 2011	Retrospective chart review	Scotland	School and Healthcare setting	86,769 girls aged 12–13 years, and 139,742 aged 13–17	Year of delivery, deprivation, entitled to free school meals (FSM)	Low	OR
2012	Donadiki et al. (27)	Greece	September 2010 to October 2011	Cross-sectional survey	Higher education Institutes in Athens: seven Universities and two Technological Educational Institutes	Healthcare setting	3,153 women aged 18–26 years	Age, educational level, smoking status, employment status, relationship status, parent’s educational status, accessibility to health care services, use of condom	Low	OR
2012	Stöcker et al. (28)	Germany	September– December 2010	Cross-sectional survey	10^th^ grade school students in Berlin	Healthcare setting	238 girls aged 14–18 years	Age, negative attitude toward vaccination in general	High	OR
2013	Bertaut et al. (29)	France	October 2010 to May 2011	Cross-sectional survey	Middle and high schools in the Department of Côte d’Or, France	Healthcare setting	948 girls aged 14–19 years	School status, school area, physician’s recommendation, mother socioeconomic status, father socioeconomic status, composition of the family, tobacco use, talk about sexuality with parents	Moderate	OR
2013	Spencer et al. (30)	UK	2011	Retrospective chart review	North-West of England	PCTs	117,343 girls aged 12–16 years	Mother’s cervical screening attendance, abnormal cervical screening history, eligibility for screening	Low	OR
2014	Mollers et al. (31)	Netherlands	2010	Cross-sectional survey	Netherlands	Healthcare setting	2,989 girls aged 16–17 years	Degree of urbanization, alcohol consumption, ever had sex, participation of mother to cervical cancer screening, program, religion	Low	OR
2012	Steens et al. (32)	Netherlands	2009	Retrospective chart review	Netherlands	Healthcare setting	337,368 girls aged 13–16 years	Mother’s screening, participation, socio-economic status, urban/rural area, ethnicity	Low	OR

*MMR, measles, mumps, and rubella; DT, diphteria, tetanus; CMU, complementary social welfare healthcare program; PCT, primary care trust; OR, odds-ratio; RRR, relative risk ratio; HR, hazard risk*.

Most of the studies reported their results based on tests for associations between variables and the following assessment of the strength of the associations. The effect measures most commonly employed were: odds-ratio (*n* = 14), relative risk (*n* = 2), and hazard ratio (*n* = 2). Other studies (*n* = 5) reported correlation coefficients and *p*-values to measure association, which also were included in the results section.

### Data variables and statistical analysis

The data analysis was performed by tabulating the data collected from the variables of interest and outcomes (HPVV uptake, either initiation, completion, or both) of the selected studies. If available, adjusted results were preferred to do the comparison among studies; however, unadjusted results were also considered in case no adjustment was performed in the study.

## Results

**Figure 1:**
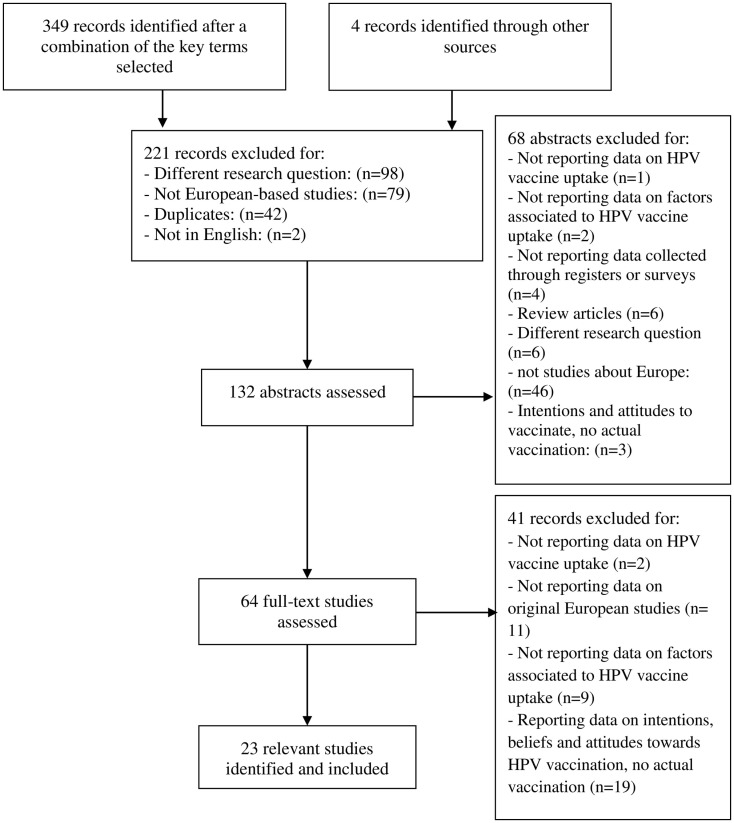
**Flow chart of study selection procedure**.

A total of 349 articles were identified through the different databases. Of these, 221 were excluded for different reasons: having a different research question (*n* = 98), not being European-based studies (*n* = 79), being duplicates (*n* = 42), or not being in English (*n* = 2). The abstracts of the remaining 128 articles were reviewed, and 68 were excluded for not being a study based in Europe (*n* = 46), for being review articles (*n* = 6), for addressing a different research question (*n* = 6), for not reporting data collected through registers or surveys (*n* = 4), for explaining intentions and attitudes toward HPVV, not actual vaccination (*n* = 3), for not reporting data on factors associated to HPV vaccine uptake (*n* = 2), and for not reporting data on HPV vaccine uptake (*n* = 1). Overall, 60 full-text studies were assessed. After assessing the inclusion and exclusion criteria, 19 were identified as relevant articles for the systematic review and 41 were discarded. The reasons for exclusion were reporting about intentions, beliefs, or attitudes toward HPVV (*n* = 19), for not reporting data on original European studies (*n* = 11), for not reporting data on factors associated to HPV vaccine uptake (*n* = 9), and finally for not providing data regarding HPV vaccine uptake (*n* = 2). Additionally, four studies were found through the reference list of some of the selected articles and included in our systematic review. Overall, 23 articles reporting HPV vaccine uptake and factors associated to it were selected since they fulfilled the inclusion criteria (Figure 1).

### Study characteristics

Overall, data on 6,247,077 women aged from 12 to 44 years were included in the studies presented in 23 articles, representing ten countries. Of the included studies, 11 focused on the HPVV initiation (10, 12–14, 18, 19, 21–23, 28, 31) and factors associated with it, three focused on HPVV completion (11, 25, 27), and nine focused on both initiation and completion (15–17, 20, 24, 26, 29, 30, 32), along with factors associated with it. Sample size ranged from 238 to 2,493,698 women, and 19 studies had a sample size over 1,000. The included studies were mostly retrospective chart reviews or retrospective cohort studies based on data extracted from registers or databases (*n* = 14). The rest were cross-sectional surveys (*n* = 6) and prospective cohort studies (*n* = 3).

## Factors Associated with Vaccine Initiation

### Ethnicity

Overall, 12 studies (10, 12, 17, 19–24, 28, 31, 32) compared HPVV initiation by ethnicity. Nine (10, 12, 17, 20–24, 32) found an association between ethnicity and HPVV initiation, where the uptake was substantially lower in areas with high ethnic minority populations. Out of the nine, three studies (20, 23, 24) conducted in the UK showed that the probability of Non-white girls being vaccinated was lower compared to White girls. The last study (32) conducted in the Netherlands found that having Moroccan ethnicity contributed to non-participation in the HPV immunization program compared with the ethnic majority in that country. That was confirmed by another study conducted in the Netherlands (32). On the contrary, three studies (19, 28, 31) [two of them (19, 28) with a very small sample size] found no association of HPVV initiation with ethnic background (Table 2).

**Table 2:** **Ethnic background as a determinant of HPVV initiation and completion**.

Authors		Initiation		Completion	
		Routine group	Catch-up group	Routine group	Catch-up group
		OR/HR 95% CI	OR/HR 95% CI	OR/HR 95% CI	OR/HR 95% CI
Rondy et al. (10)^a^^,^ ^b^ (OR)	Netherlands–Netherlands	1 (Ref.)	NR	NR	NR
	Netherlands–Surinam^c^	0.83 (0.71–0.97)	
	Netherlands–Turkey^c^	0.78 (0.64–0.97)	
	Netherlands–Morocco^d^	0.55 (0.43–0.72)	
	Surinam–Surinam^c^	0.83 (0.75–0.93)	
	Turkey–Turkey^d^	0.61 (0.56–0.66)	
	Morocco–Morocco^d^	0.33 (0.31–0.37)	
Widgren et al.(12)^e^ (HR)	Danish-born w both parents Danish-born	1 (Ref.)	NR	NR	NR
	Danish-born w one parent Danish-born	0.84 (0.79–0.89)	
	Danish-born w none of parents Danish-born	1.02 (0.98–1.06)	
	EU/EFTA country. non-Danish	0.74 (0.67–0.82)	
Blödt et al. (19) (OR)	No migration background	NS^f^	NR	NR	NR
	Migration background	
Fisher et al. (20)^g^ (OR)	White British	1 (Ref.)	NR	1 (Ref.)	NR
	Mixed ethnicity	0.94 (0.55–1.61)^h^		NS	
	Asian or British Asian	0.59 (0.44–0.80)^i^		NS	
	Black or British Black	0.50 (0.32–0.79)^i^		NS	
	Chinese and other	0.48 (0.33–0.71)^j^		NS	
	Non-stated	0.44 (0.39–0.50)^j^		0.77 (0.65–0.92)^i^	
Kumar et al. (22)	White	NR^k^	NR^k^	NR	NR
	Asian	
	Black	
	Other	
Roberts et al. (23)^l^ (OR)	White	1 (Ref.)	NR	NR	NR
	Other	0.72 (0.52–0.99)^c^	
Spencer AM et al. (24)^j^^,^ ^m^ (OR)	White	1 (Ref.)	1 (Ref.)	NS	1 (Ref.)
	Mixed	0.73 (0.53–1.02)	1.28 (0.94–1.75)		0.51 (0.33–0.79)
	Asian	0.90 (0.88–0.92)	0.95 (0.93–0.97)		1.06 (1.02–1.09)
	Black	0.85 (0.77–0.94)	0.79 (0.71–0.85)		1.05 (0.91–1.21)
	Other	0.65 (0.51–0.83)	0.70 (0.56–0.89)		0.72 (0.52–1.00)
Stöcker et al. (28) (OR)	No migration background	NS	NR	NR	NR
	Migration background	
Mollers et al. (31) (OR)	Dutch	NS	NR	NR	NR
	Non-Dutch	
Brabin et al. (21)	British	NR^o^	NR	NR	NR
	Ethnic minorities	
Steens et al. (32) (OR)	Dutch	1 (Ref.)	NR	NR	NR
	Moroccan	6.6% (6.1–7.0)^n^	
Ganry et al. (17)		NR^p^	NR	NR^q^	NR

*NR, not reported*.

*^a^Country of birth of parents*.

*^b^Adjusted on implementation aspects and dates of vaccination*.

*^c^*p*-value <0.05*.

*^d^*p*-value <0.0001*.

*^e^Adjusted for place of residence (area), urban/rural (population density), place of origin, age of mother, and number of siblings*.

*^f^Results: 44 vs. 70% (HPVV uptake by girls with migration background vs. girls without migration background); *x*^2^ = 4.44; *p* = 0.04*.

*^g^Adjusted for ethnicity, deprivation quintile, primary care trust (PCT)/local authority, program year, and educational setting*.

*^h^NS, non-significant*.

*^i^*p*-value <0.005*.

*^j^*p*-value <0.001*.

*^k^Association between ethnicity and HPVV uptake: final regression results: *r*^2^ = 0.25 for the routine group and *r*^2^ = 0.08 for the catch-up group*.

*^l^Adjusted for index of deprivation score (ID score)*.

*^m^Adjusted for PCT (primary care trust) level and IMD (index of multiple deprivation)*.

*^n^About 6.6% of non-participation in cervical cancer screening and HPV immunization was attributed to the fact that the girls had Maroccan ethnicity compared to Dutch girls*.

*^o^The results show a significant lower HPVV uptake in schools with a high number of girls belonging to ethnic minorities in the UK (*p*-value <0.001 for trend)*.

*^p^Negative correlation between HPVV initiation and rate of immigrants (*r*^2^ = 0.06; *p*-value = 0.007)*.

*^q^Negative correlation between HPVV completion and rate of immigrants (*r*^2^ = 0.1; *p*-value <0.001)*.

*The results are reported as OR (95% CI), HR (95% CI), or RR (95% CI)*.

### Socio-economic status and education level

Fourteen articles (10, 13–15, 17, 20–24, 26, 29, 31, 32) reported data on socio-economic status or area-level indicators and HPV vaccine initiation. All studies (10, 13–15, 21–24, 26, 29, 32) but three (17, 20, 31) showed an association between pertaining to a disadvantaged socio-economic group and lower HPVV initiation. For two of these studies (22, 24), however, this association was only seen for those girls that belong to the catch-up group (17–18 and 14–16 years, respectively). On the contrary, three studies (17, 20, 31) found no evidence of association between HPVV initiation and socio-economic status. Regarding education level as such, a small study (19) found that girls with more than 11 years of school education had a higher HPVV initiation than those with less than 11 years of education. These results were supported by another study (14) that found that girls enrolled in a high school or higher degree had higher vaccine initiation than those with a lower degree (Table 3).

**Table 3:** **Socio-economic status (SES) and education level as a determinant of HPVV initiation and completion**.

Authors	Definitions of SES	OR/HR/RR 95% CI
**Initiation**
Mollers et al. (31) (OR)	**SES** is defined as the average income per household in a given postcode area with percentage of households with low income, without a paid job, and with low average education. That results in a score range. The lower the score, the higher the socio-economic status.	NS^a^		
Giambi et al. (14)^b^ (OR)	**Employment status** divided in three groups: Unemployed, housewife, other (reference) Employed Student	1 (Ref.) 1.12 (0.76–1.63) 1.64 (1.13–2.37)		
	**Education level** divided in two groups: Primary or middle school degree (reference) High school degree	1 (Ref.) 1.41 (1.02–1.93)		
Brabin et al. (21)	**Schools with high proportion of girls entitled to free school meals (FSM)**	NR^c^		
Kumar et al. (22)	**Material deprivation** was measured following the Index of Multiple Deprivation (IMD) of 2007	NR^d^		
Rondy et al. (10)^e^ (OR)	**SES:** defined as the average income per household in a given postcode area with percentage of households with low income, without a paid job, and with low average education. That results in a score range. The lower the score, the higher the socio-economic status	Results from a multilevel analysis: SES (score *n −* 1 vs. *n*): 1.05 (1.03–1.06)^f^		
Lefevere et al. (13) (HR)	**The median income of the neighborhood** where the girls live in is divided in quintiles: quintile 1 to quintile 5 (from the most to the least deprived areas)	Quintile 1: 0.75 (0.72–0.77) Quintile 2: 0.93 (0.90–0.95) Quintile 3: 1 (Ref.) Quintile 4: 1.04 (1.02–1.07) Quintile 5: 1.10 (1.07–1.12)
	**Right to preferential treatment (No**-reference/**Yes)**: it means that the group with the right to preferential treatment pays lower copayments. It depends on whether the household income is below a certain threshold	1 (Ref.) 0.55 (0.52–0.58)		
Roberts et al. (23)^f^^,^ ^g^ (OR)	**Deprivation:** lower super output areas (LSOA) and the corresponding Index of Deprivation 2007 were obtained. The sample was divided by quintiles (2). Quintile 1 (most deprived) to Quintile 5 (least deprived)	Index of multiple deprivation Per 10-point increase: 0.80 (0.85–0.95)		
Ganry et al. (17)	**Socio-economic status** was calculated through “*the median income per consumption unit and the percentage of taxable households”* (20)	NS^h^		
Steens et al. (32)^i^ (OR)	**SES** is defined as the average income per household in a given postcode area with percentage of households with low income, without a paid job, and with low average education. That results in a score range. The lower the score, the higher the socio-economic status	Results from multilevel analysis: Area with low SES: 7.6% (7–8.2%) Area with moderate–low SES: 6.4% (5.5–7.3%)		
Blödt et al. (19) (OR)	**Education level: years of school education:** <11 years/≥11 years (ref.)	0.45 (0.20–1.02) 1 (Ref.)		
**Completion**
Hughes et al. (25)	**SES** is divided in groups of areas according to deprivation level: Q_1_ (most deprived) to Q_5_ (least deprived)	NR^j^		
**Initiation and completion**
Lions et al.(15)^k^ (RR)	**CMU beneficiary** (No/Yes) Usually, the 65% of the total HPV vaccine price is reimbursed by the Social Security in France. But for patients covered by the complementary social welfare healthcare program (CMU) (an indicator of lower socio-economic status), 100% vaccine of the price is reimbursed	**Initiation:** 1 (Ref.) 0.71 (0.68–0.75)	**Completion:** 1 (Ref.) 0.78 (0.76–0.81)	
Fisher et al. (20) (OR)	**Deprivation:** “postcodes from individual records were linked to the corresponding lower super output areas (LSOA) and deprivation score was assigned using the Index of Multiple Deprivation of 2010, and the sample analyzed as quintiles” (20). Quintile 1 (most deprived) to Quintile 5 (least deprived)	**Initiation:** NS^l^	**Completion:** NS^l^	
Sinka et al. (26) (OR)	Results based on the association of each vaccination record to a Scottish Index of **Multiple Deprivation** (SIMD) quintile, which uses the postcode of residence. SIMD1 = the most and SIMD5 = the least deprived areas	**Initiation:** 1 (most deprived) – (Ref.); 1.25 (1.14–1.36); 1.39 (1.27–1.53); 1.59 (1.44–1.75); 1.80 (1.63–1.99) (least deprived)		**Completion:** 1 (most deprived) – (Ref.); 1.28 (1.20–1.37); 1.54 (1.43–1.65); 1.83 (1.70–1.97); 2.15 (1.99–2.31) (least deprived)
	**FSM:** % of girls eligible for free school meals at school. FSM-1 (least) to FSM-5 (most)	**Initiation:** 1 (least) – (Ref.); 1.07 (0.80–1.43); 1.32 (0.99–1.77); 0.98 (0.73–1.31); 0.80 (0.61–1.04) (most)		**Completion:** 1 (least) – (Ref.); 1.03 (0.80–1.32); 1.16 (0.90–1.49); 0.80 (0.62–1.04); 0.75 (0.60–0.94) (most)
Bertaut et al. (29)^m^ (OR)	**Parents SES** are divided into three categories according to family income: Under-privileged (ref.) Medium Privileged	**Initiation:** mother SES^n^: 1 (Ref.); 1.5 (1.1–2.1); 1.6 (1.1–2.4)	Father SES^o^: 1 (Ref.); 1.4 (0.8–2.2); 1.7 (1.3–2.2)	**Completion:** mother SES: NS;	Father SES^p^: 1 (Ref.); 0.8 (0.4–1.4); 0.4 (0.2–0.8)
Spencer et al. (24)^o^ (OR)	**Deprivation** is defined as “the Index of Multiple Deprivation (IMD) 2010 associated with the LSOA derived from the address postcode (24)”. “The deprivation indices derive from a combination of measures of income, employment status, disability, health, crime, education, barriers to housing and services, and living environment from the UK census of 2010.” The results are given divided in quintiles. Quintile 1 (most deprived) to Quintile 5 (least deprived-reference) (24)	**Initiation:** routine group: NS	Catch-up group: 0.75 (0.63–0.88); 0.75 (0.65–0.88); 0.91 (0.78–1.01); 1.04 (0.89–1.21); 1 (Ref.)	**Completion:** routine group: 0.75 (0.63–0.88); 0.75 (0.65–0.88); 0.91 (0.78–1.01); 1.04 (0.89–1.21); 1 (Ref.)	Catch-up group: 0.64 (0.57–0.71); 0.77 (0.70–0.86); 0.91 (0.82–1.01); 1.02 (0.91–1.13) 1 (Ref.)

*^a^*p*-value = 0.2*.

*^b^Results from a multivariate logistic regression model, where all variables with a *p*-value <0.10 in the univariate model were included*.

*^c^The study shows that HPVV uptake was significantly lower in schools with a higher proportion of students entitled to FSM (*p* = 0.029)*.

*^d^Results not reported as OR/HR. Regression results for multiple deprivation show that deprivation was a salient factor for uptake [*B* = −2.76% (−5.27 to −0.24); adjusted *r*^2^ = 0.08] and fall in uptake [B = 1.82% (0.28–3.35); adjusted *r*^2^ = 0.12] in the catch-up group*.

*^e^Adjusted on implementation aspects and dates of vaccination*.

*^f^*p*-value <0.0001*.

*^g^Adjusted for ethnicity*.

*^h^No correlation was found between socio-economic factors and HPVV coverage (*p* = 0.6 and *p* = 0.8)*.

*^i^The results from a multivariable multilevel logistic regression analysis show that 7.6% of non-participation in cervical cancer screening and HPV immunization was attributed to the fact that the girls lived in a neighborhood with a moderate-low SES compared to if these girls would have been living in a neighborhood with high SES*.

*^j^Estimation of HPV vaccine coverage by deprivation level. Results of the percentage vaccination coverage mean (95% CI) from the most deprived to the least: 26.5 (23.3–29.6), 33.3 (29.6–37.0), 35.4 (30.8–40.0), 43.9 (40.5–47.2), 41.7 (38.4–45.0), 37.4 (35.7–39.1). *p*-value for trend (Wald test) <0.001*.

*^k^Results from multivariate analysis*.

*^l^The results were not significant in the multivariable model (*p*-value = 0.48)*.

*^m^Results adjusted for school status and school area*.

*^n^*p*-value = 0.019*.

*^o^*p*-value <10^−3^*.

*^p^*p*-value <10^−4^*.

*The results are reported as OR (95% CI) or HR (95% CI)*.

### Age

Of the seven studies including a cohort of girls aged 12–24 years (10, 13, 16–18, 26, 28), which allowed comparison of HPVV initiation between different ages, three showed the highest vaccination initiation at the age of 16–18 years (13, 16, 28), two (10, 17) at the age of 14–15 years, one (18) at the age of 14–16 years, and one (26) showed similar HPVV initiation from 12–17 years of age. Additionally, two studies including a cohort of girls aged 18–26 years (14, 19) found no significant association between age and HPVV initiation (Table 4).

**Table 4:** **Age as a determinant of HPVV initiation and completion**.

Outcome	Age					
	<13	14–15	16	17–18	19–24	>25
**Initiation**
Rondy et al. (10)^a^ (OR)	1.06 (1.03–108)	1.11 (1.09–1.14)	Ref.	NR	NR	NR
		1.10 (1.08–1.12)	
Lefevere et al. (13)^b^ (HR)	0.23 (0.20–0.25)	0.61 (0.55–0.67)	4.32 (4.11–4.55)	11.74 (10.89–12.65)	NR	NR
	0.21 (0.19–0.24)	Ref.		19.39 (17.47–21.52)	NR	
	0.37 (0.33–0.41)					
Giambi et al. (14)^c^ (OR)	NR	NR	NR	NR	NS	NS
Blödt S et al. (19)^d^ (OR)	NR	NR	NR	NS	NS	NR
Stöcker et al. (28)^e^ (OR)	NR	–	–	2.19 per year of life (1.16–4.15)	NR	NR
**Completion**
Lions et al. (15)^f^ (RR)	NR	NR	Ref.	1.06 (1.02–1.10)	NR	NR
Donadiki et al. (27)^g^ (OR)	NR	NR	NR	1.24 (1.02–1.50)	Ref.	NR
**Initiation**
Rouzier et al. (16)^h^	NR	18, 30%	32%	29, 26%	16, 8, 5, 4, 4%	NR
Lutringer-Magnin et al. (18)^i^	NR	NR	68.2%	56.9%	18.7%	NR
Sinka et al. (26)^j^	93.7%	94.6%	93.0, 48.5%*	NR	NR	NR
Ganry et al. (17)^k^	0.3%	22.3, 25.8%	16.2%	13.6, 9.3%	5.2, 2.9, 1.8, 1.4, 1.1, 0%	
**Completion**
Rouzier et al. (16)^l^	NR	NR	NR	45.8, 34%	NR	NR
Ganry et al. (17)^m^	NR	65.5, 43.1%	NR	NR	NR	NR
Sinka et al. (26)^j^	89.4%	89.9%	86.6, 31.8%*	NR	NR	NR

*NR, not reported/NS, non-significant*.

*^a^Age group: year of birth: 1994, 1995, 1996. Adjusted for implementation aspects and dates of vaccination*.

*^b^Age group: year of birth: 1989, 1990, 1991, 1992, 1993, 1994, 1995, 1996. Results from a Cox regression model*.

*^c^Age group: 18–24 years of age, 25–26 years of age. Not significant association between age an HPVV uptake after the multivariate logistic regression model*.

*^d^Age group: 18–20 years, 21–25 years. Results from the multivariate analysis*.

*^e^Age group: 14–18 years of age. 91.3% of the females were 15 years old or older. Girls that were vaccinated, were more likely to be older. *p*-value = 0.02*.

*^f^Age group: <17 years of age, ≥17 years of age*.

*^g^Age group: 18–20 years of age, 21–26 years of age. Results from multivariate logistic regression*.

*^h^Results from correlation coefficients and *p*-values: maximum coverage rate at the age of 16. From 16 onward, coverage rate decreases (*p* < 0.001)*.

*^i^Maximum HPVV initiation rate at the age of 14–16 years (68.2%), followed by 17–20 (56.9%) and 21–23 (18.7%). A backward logistic regression was performed with the group of girls aged 14–18 years. Age was embedded in the multivariate analysis*.

^j^HPVV uptake in Scotland in 2008. Age group: 12–13 years of age (routine cohort), 15–16 and 16–17 years of age (catch-up cohort: divided into those that are at school and those that already left it*).

^k^Age group: 13–14* years of age, (*routine), 15–23 years of age (catch-up).

*^l^Results from correlation coefficients and *p*-values: Maximum completion rate under 18 years of age (*p* < 0.0001)*.

*^m^Among the older group (catch-up), those aged 15 years at the time of the first dose are more compliant than older girls*.

### Vaccination history

Having received, previous childhood immunization was associated with higher HPVV initiation in all studies (10, 12, 20, 23) that compared immunization initiation by childhood vaccination history, but one small study (28). Being vaccinated against Hepatitis B was found to be associated with higher HPVV initiation in one study (18) (see Table S1 in Supplementary Material).

### Mother’s cervical screening attendance

Regular participation of mother in cervical cancer screening programs was positively associated with HPVV initiation in four studies (18, 30–32) that compared uptake by mother’s screening attendance. In one of the studies (32), the likelihood of HPVV initiation was 40% higher if the mother regularly participated in cervical cancer screening programs compared to those whose mothers did not participate (see Table S2 in Supplementary Material).

### Area of residence

One Danish study (12) making reference to population density showed that girls living in the least urbanized areas (1–9 inhabitants/km^2^) had the lowest HPVV initiation (aHR = 0.87; 95% CI: 0.77–0.97) compared to the most urbanized areas (>1,000 inhabitants/km^2^). HPVV initiation in urban schools was twice that of rural schools (aOR = 1.9; 95% CI: 0.86–0.99) in another study (29) conducted in France. This was supported by another study also conducted in France (15), which found an association between living in a rural area and having a lower HPVV initiation (aOR = 0.96; 95% CI: 0.92–0.99). On the contrary, living in rural areas (<1,000 inhabitants/km^2^) remained associated with higher vaccine initiation in two other studies conducted in the Netherlands [aOR = 0.80; 95% CI: 0.70–1.00 for a high degree of urbanization (31) and 6.6%; 95% CI: 6.10–7.00 for the other study (32)]. This result means that looking at the participation in HPV immunization programs and cervical cancer screening, 6.6% of non-participation in both prevention programs was attributed to the fact that they were living in an urban area (32).

### Consultation with a specialist or physician’s recommendation

Having consulted with a specialist (pediatrician, family physician, gynecologist, or other) was associated with higher vaccination initiation in one study (aRR = 1.33; 95% CI: 1.30–1.36) (15) and physician’s vaccine recommendation was associated with HPVV initiation in another study (29) (aOR = 2.8; 95% CI: 1.70–4.70).

### Other factors

One study showed a lower uptake for those girls who never had sexual intercourse in the past (OR = 0.44; 95% CI: 0.17–1.11) (19), whereas the other revealed that having had sex in the past is a negative predictor of HPVV initiation (aOR = 0.80; 95% CI: 0.60–1.00) (31).

## Factors Associated with Vaccine Completion

### Ethnicity

Lower HPVV completion was observed among ethnic minorities in the catch-up group in a study (24) that compared vaccine completion in the routine and catch-up group by ethnicity. This was supported by another study (17) that found a negative correlation between having completed vaccination by ethnic minorities (*r*^2^ = 0.1; *p* < 0.001). Another study (20) also found an association between belonging to an ethnic minority group and lower HPVV completion, but in this case, only those girls belonging to the ethnic category “non-stated” showed lower vaccine program completion (aOR = 0.77; 95% CI: 0.65–0.92; *p* < 0.004). The study defined the “Non-stated” category as a group consisting of either children of populations that had missing ethnicity because they were born outside the participating Primary Care Trust (PCT) (e.g., immigrants), because parents did not want to reveal their ethnicity prior to the child’s birth, or because parents did not understand that specific question at the moment they were asked (Table 2).

### Socio-economic status

Four studies (15, 24–26) out of six (15, 20, 24–26, 29) reporting information about the three-dose program completion and socio-economic status showed an association between non-completion and low socio-economic background. However, for one of these studies (25), the negative association was only observed among the oldest group of girls (aged 16 or older). In contrast, one study (20) found no association between lower HPVV completion and deprivation (*p* = 0.48), and one study (29) showed that after initiation of the vaccination program, girls who attended private schools [aOR = 0.50 (95% CI: 0.40–0.80), *p* < 0.001] or who belonged to families where the father has higher incomes were less likely to complete the three-dose program. Girls with at least one university-educated parent were more likely to be fully vaccinated than those whose parents did not finish high school [relative risk reduction (RRR) = 15.45; 95% CI: 14.65–16.30] (11). The same trend was seen in another study (27), which also compared vaccine completion between University and Technological Educational Institute students, and the results showed a higher program completion among University students (OR = 1.22; 95% CI: 1.01–1.49) (Table 3).

### Age

Two studies (15, 27) reporting vaccine completion by age group showed that girls aged over 17 years were more likely to complete the vaccination program compared to other groups of age. Two other studies (16, 17), however, revealed that girls under 18 years of age were more likely to complete it. More specifically, one of these studies (17) showed higher program completion in girls aged 13–14 years. On the contrary, a study conducted in Scotland (26) found high vaccine program completion among all girls in that study, except from the group of girls aged 16–17 years that had already left school, where the uptake was lower (Table 4).

### Mother’s cervical screening attendance

Two studies (30, 32) found positive associations between mothers’ cervical screening attendance and girls’ program completion [aOR = 2.2; 95% CI: 1.6–2.9, and aOR = 1.54; 95% CI: 1.51–1.57, respectively] (see Table S2 in Supplementary Material).

### Area of residence

One study (15) reporting data on HPV vaccine completion by rural/urban area showed a lower vaccine completion in girls living in rural areas (RR = 0.92; 95% CI: 0.86–0.98) compared to those living in urban areas, whereas another study (29) did not find significant variation in the results by population areas (*p* = 0.24).

### Prescriber

Two studies (15, 17) found an association between the specialization of the physician responsible for the vaccine prescription and the completion of the vaccination program. While one study (17) found that the program completion was higher if the prescriber of the first dose was a pediatrician or a gynecologist compared to a general practitioner [48.1, 44.7, and 38.3%, respectively (*p* < 0.001)], another found that the highest program completion was achieved if the prescriber was a family physician [(RR = 0.90; 95% CI: 0.86–0.94) for the gynecologist, and family physician was the reference group] (15). A third study (16) found no significant correlation between prescriber (general practitioner vs. gynecologist) and HPVV program completion.

## Discussion

### Main findings

Belonging to ethnic minority groups and having a disadvantaged socio-economic status were associated with lower HPVV initiation and completion in the majority of the studies. The highest HPVV program initiation was observed at the age of 16–18 years in more than half of the studies. Consultation with a specialist was associated with higher HPVV initiation. Regular cervical screening participation by the mother was associated with higher HPV vaccine program initiation and completion. Having received previous childhood vaccinations was associated with higher HPVV uptake.

### Findings in relation to other studies

Regarding ethnicity, there is an overall association between belonging to an ethnic minority group and having a lower probability of HPVV uptake. That suggests that cultural factors concerning sexually transmitted infections could be an important issue in the HPV vaccine uptake. This result is similar to the previous systematic review mostly based on American studies that showed lower likelihood to initiate HPVV program among ethnic minority groups compared to the ethnic majority population (8). Two of the three studies that did not report a significant difference in uptake between ethnic backgrounds had a population sample inferior to 500 girls. Thus, the number of ethnic minority girls participating in the study might not be large enough to be representative.

Most of the studies showed an association between higher deprivation level and lower HPVV program initiation and completion. This is especially relevant since women and girls with low socio-economic status are at a special risk of developing cervical cancer (33, 34). Taking into account that the HPVV is offered for free in the majority of the European countries, the lower uptake among deprived people is unlikely to be a result of purely economic reasons. Studies (35–39) reporting data on social inequalities in healthcare indicate lower healthcare participation among socioeconomically deprived populations. This indicates that the lower vaccine uptake among most deprived populations might be explained by a combination of factors; parents and girls may have a different perception on the importance of HPVV as a preventive measure, parents and girls may not have received information about the immunization program, or if received, did not have the time to read it or the knowledge or language skills to understand it (39, 40). Some of the studies (22, 24, 25) measuring completion rates by socio-economic status, however, showed lower immunization program completion only for the catch-up group. In this case, the reason is likely to be the different delivery mechanism employed for older girls. The majority of girls in the routine cohort received the vaccination at school, whereas older girls are often sent to healthcare settings to receive the vaccine. This can lead to a decrease in adherence to the three-dose immunization schedule since the girls or the parents have to arrange an appointment at the clinic instead of having arranged it by the school and during normal school hours.

As referred by Koulova et al. (5) and Garnet et al. (41), it is recommended to start the vaccination program at a younger age and preferably before sexual onset in order to increase the vaccine effectiveness. However, in the light of our results, older adolescents have a higher probability to initiate and complete the program. The highest vaccination initiation rate was seen at the age of 16–18 years in four out of seven studies. These results are comparable to those published in a systematic review (4), which shows that higher vaccination rates were achieved among older adolescents. According to Dempsey et al. (42), one reason could be the fact that parents might be more likely to accept the vaccination as the age of the daughter increases.

According to the literature (43, 44), parental attitudes toward preventive measures often influence the decision-making of their daughters. This suggests that parents’ general perceptions toward vaccinations may play a role also with regard to the uptake of HPVV. Furthermore, regular mothers’ screening attendance has been found to be associated with HPVV program initiation and completion in a variety of studies in the literature (45–47). This suggests that mothers who attend preventive health services acknowledge their relevance, and may transmit these positive attitudes to their daughters or to decisions regarding their daughters, which positively contributes to a high HPVV uptake. It is a concern that non-attending cervical screening is a predictor for under-vaccination, which suggests that there may be less added value of vaccination.

### Limitations

There are several potential limitations. There is a risk of selection bias, which was assessed in the different studies according to sample size and the presence or absence of adjustment for potential confounders. Studies reporting data of small samples (<1,000 participants) were considered to be at moderate-to-high risk of bias (Table 1). Studies were heterogeneous in the study design, in the independent variables included, and in the definition of reference groups, making it difficult to compare results. Additionally, vaccination coverage was reported by either caregivers, by reimbursement data, or by the people themselves, introducing a risk of misclassification bias, recall bias, or response bias. Sampling strategy also differed across studies, leading to a potential selection bias. Some studies lacked statistical significance, given the small size and given that no adjustment for potential confounders was performed. Additionally, there was little consistency in the factors controlled for in the analysis across studies, limiting potentially the comparison of the study results. Most of the studies were performed in rather wealthy countries, and no study was found from Central- or Eastern-Europe, where the majority of cervical cancer cases occur, even though the vaccine is available in most of the European countries.

## Conclusion

We found an association between ethnic minority background and disadvantaged socio-economic status and lower HPVV uptake in Europe. Given that the vaccine is offered for free in most of the European countries, the findings suggest that ethno-cultural and educational factors may be important when it comes to HPVV uptake. The fact that girls who are undervaccinated with HPVV also have lower uptake of standard childhood vaccines and mothers that are less likely to attend cervical cancer screening indicate that the reasons for non-vaccination are related to a general lower compliance with preventive health rather than specific concerns about HPVV. Efforts should be put into providing vulnerable populations with a targeted information on the vaccine, and health interventions such as vaccination campaigns should specifically target them to improve HPVV uptake.

Because higher effectiveness is achieved if the vaccine is administered prior to sexual onset, communication efforts should be made to increase the HPVV acceptance among young adolescents.

Since some girls leave school early, and therefore do not take part of the school vaccination programs, a reminder program can be designed where a letter is sent to each of the girls missing any dose, aimed at reaching the maximum HPVV program initiation and completion.

Further similar studies in other European countries, especially in Eastern and Central Europe, are needed to get a representative population and to find determinants in the HPVV uptake in these countries. The creation of patterns in the HPVV uptake would allow targeting these populations in which the uptake is significantly lower, with the ultimate objective of reducing the cervical cancer burden.

## Author Contributions

VFC and LC-A contributed to the conception, design, and drafting of the study. VFC was responsible for acquisition and analysis of data. VFC, LC-A, and JGC contributed to the analysis and interpretation of data. VFC, LC-A, and JGC revised it for critically important intellectual content. All authors have seen and approved the final version submitted.

## Conflict of Interest Statement

The authors declare that the research was conducted in the absence of any commercial or financial relationships that could be construed as a potential conflict of interest.

## Supplementary Material

The Supplementary Material for this article can be found online at http://journal.frontiersin.org/article/10.3389/fonc.2015.00141
