# Supplementary material for: Determinants in the Uptake of the Human Papillomavirus Vaccine: A Systematic Review Based on European Studies
Source: Front Oncol. 2015 Jun 24;5:141. doi: 10.3389/fonc.2015.00141 (PMC4478848; doi:10.3389/fonc.2015.00141)
Supplement: Supplementary file 1 [file Table_1.DOCX]

**Supplementary file 1**. Vaccination history as a determinant of HPV vaccination

| **Authors** |  |  |  |  |  |
| --- | --- | --- | --- | --- | --- |
| **Outcome** |  | MMR | MMR2 | DT | Hepatitis B |
|  |  | OR/HR 95%CI | OR/HR 95%CI | OR/HR 95%CI | OR/HR 95%CI |
| Rondy M et al^17,a^ (OR) |  | NR | 6.26 (5.87-6.68) | NR | NR |
| Widgren K et al^22^ (HR) |  | NR | 2.39 (2.29-2.49) | 1.54 (1.49-1.60) | NR |
| Lutringer D et al^10^ |  | NR | NR | NR | 3.2 (1.6-6.1) |
| Fisher H et al^28^ (OR) |  | 3.64 (3.27-4.04) | NR | NR | NR |
| Roberts SA et al^19^ (OR) |  | 1.89 (1.17-3.02) | 1.33 (1.02-1.72) | NS | NR |
| Stöcker P et al^12^ (OR) |  | NS | NS | NS | NR |

MMR: Measles-Mumps-Rubella (first dose)

MMR2: Measles-Mumps-Rubella (second dose)

DT: Diphteria-Tetanus

NR: Not reported/ NS: Not significant

^a^ Adjusted for implementation aspects and dates of vaccination
