# Supplementary material for: Determinants in the Uptake of the Human Papillomavirus Vaccine: A Systematic Review Based on European Studies
Source: Front Oncol. 2015 Jun 24;5:141. doi: 10.3389/fonc.2015.00141 (PMC4478848; doi:10.3389/fonc.2015.00141)
Supplement: Supplementary file 2 [file Table_2.DOCX]

**Supplementary file 2**. Mother´s cervical screening attendance as a determinant of HPV vaccine uptake

| **Authors** |  | **Mother´s cervical cancer screening** | |
| --- | --- | --- | --- |
| Outcome |  | OR 95%CI | |
| **Initiation**  Mollers M et al^13,a^ | No  Yes  Unknown | 1  1.6 (1.2-2.0)  1.3 (1.0-1.6)  Routine group | Catch-up group |
| Spencer (nee Pikington) et al^25,b^ | No  Screened but not in the las 5 years  Screened in the last 5 years | 1  2.4 (2.1-2.7)  3.5 (3.1-4.0) | 1  2.1 (1.8-2.4)  3.4 (2.9-3.9) |
| Lutringer D et al^10,c^ | No  Yes  Unknown | 1  6.2 (1.5-25-8)  4.7 (0.98-22.7) |  |
| Steens A et al^32,d^ | No  Yes | 1  1.40 (1.38-1.43) |  |
| **Completion** |  |  |  |
| Steens A et al^32^ | No  Yes | 1  1.54 (1.51-1.57) |  |
| Spencer (nee Pikington) et al^25,b^ | No  Screened but not in the las 5 years  Screened in the last 5 years | Routine group  1  1.6 (1.2-2.1)  2.2 (1.6-2.9) | Catch-up group  1   - 1. (0.8-1.4)   1.5 (1.2-2.0) |

a Only variables with a p-value <0.1 in the univariate analysis were included

b Afjusted for mother´s age, Primary Care Trust (PCT) and vaccination cohort

c Adjusted Odds Ratio
